# Epidemiological Scenario of American Trypanosomiasis and Its Socioeconomic and Environmental Relations, Pará, Eastern Brazilian Amazon

**DOI:** 10.3390/tropicalmed10040088

**Published:** 2025-03-28

**Authors:** Claudia do Socorro Carvalho Miranda, Bruna Costa de Souza, Tainara Carvalho Garcia Miranda Filgueiras, João Simão de Melo Neto, Amanda Sophia Carvalho Miranda da Silva, Hilton Pereira da Silva, Marcos Valério Santos da Silva, Frederico Itã Mateus Carvalho Oliveira Miranda, Edilene do Socorro Nascimento Falcão Sarges, Sérgio Luiz Althoff, Selma Kazumi da Trindade Noguchi, Nelson Veiga Gonçalves

**Affiliations:** 1 Laboratory of Epidemiology and Geoprocessing of Amazon, University of the State of Pará (UEPA), Belém 66050-540, Brazil; bruna.souza@uepa.br (B.C.d.S.); amanda.miranda.silva@ics.ufpa.br (A.S.C.M.d.S.); frederico22250073@aluno.cesupa.br (F.I.M.C.O.M.); edifalcao@ufpa.br (E.d.S.N.F.S.); selma.noguchi@uepa.br (S.K.d.T.N.); nelsonveiga@uepa.br (N.V.G.); 2 Programa de Pós-Graduação em Saúde Coletiva na Amazônia, Federal University of Pará (UFPA), Belém 66075-110, Brazil; jsmeloneto@ufpa.br (J.S.d.M.N.); hdasilva@ufpa.br (H.P.d.S.); marcossilva@ufpa.br (M.V.S.d.S.); 3 Programa de Pós-Graduação em Inteligência Territorial e Sustentabilidade, Centro Universitário do Pará (Cesupa), Belém 66060-230, Brazil; 4 Programa de Pós-Graduação em Direito, Federal University of Pará (UFPA), Belém 66075-110, Brazil; tainara.filgueiras@ifch.ufpa.br; 5 Center for Advanced Multidisciplinary Studies, University of Brasília, Brasília 70910-900, Brazil; 6 Animal Biology Laboratory, Natural Sciences Department, Blumenau Regional University (FURB), Blumenau 89012-078, Brazil; althoff@furb.br; 7 Cyberspace Institute, Federal Rural University of Amazon (UFRA), Belém 66077-830, Brazil

**Keywords:** chagas disease, epidemiology, environment, spatial analysis, public policies

## Abstract

Chagas disease is a serious public health problem worldwide. In Brazil, the state of Pará has the largest number of reported cases. This article analyzes the spatial distribution of this disease and its relationship with socioeconomic, environmental, and public policy health variables in three mesoregions in the Pará state from 2013 to 2022. This ecological study used secondary data obtained from official Brazilian agencies. Spatial analysis was carried out using the flow, kernel, and bivariate global Moran techniques expressed in thematic maps. A total of 3664 cases of the disease were confirmed, with the highest number of cases being reported in the northeast of Pará. A seasonal pattern of the disease, an epidemiological profile similar to other diseases in the Amazon region, and the spatial dependence between the disease prevalence and socioeconomic indicators were observed. The most intense movement of patients for treatment was to the Belém metropolitan mesoregion, which has the majority of the health services and professionals. The disease showed an inhomogeneous pattern of cases in terms of the spatial distribution, with a direct relationship between areas with a higher number of cases and those with human clusters. The socioenvironmental origins of the disease transcend mesoregion boundaries and stem from the historically unsustainable development model in the Amazon.

## 1. Introduction

Chagas disease (CD), or American trypanosomiasis, is a chronic, multi-system parasitic disease caused by the hemoflagellate protozoan *Trypanosoma cruzi* (Chagas, 1909) of the order Kinetoplastida, whose main vectors are hemipteran insects of the genera *Panstrongylus*, *Rhodnius*, and *Triatoma*, belonging to the *Reduviidae* family [1,2]. This disease can be transmitted vectorially, orally, or through blood transfusions, organ transplants, and accidents involving contaminated biological materials, as well as vertically [1,2]. Currently, *T. cruzi* is found circulating in nature among hundreds of species of mammals (wild and domestic) distributed across seven different orders and dozens of species of vectors [1,3,4]. The disease has a biphasic clinical course, with an acute phase (asymptomatic or symptomatic) and a chronic phase that can manifest itself in indeterminate, cardiac, digestive, or cardiodigestive forms [1,2].

CD is part of the World Health Organization’s (WHO) group of neglected tropical diseases (NTDs). It is estimated that 6 to 7 million people are infected with *T. cruzi* in 44 countries around the world [5,6]. The disease is endemic in 21 Latin American countries, with approximately 75 million people living in areas at risk of transmission, and annual records show around 30,000 new cases and 12,000 deaths related to this disease [5,6,7]. In recent decades, it has expanded and has been reported in non-endemic areas in the United States, Canada, Europe, Africa, and Asia [8]. Its occurrence is associated with various factors, including poverty, uncontrolled human migration, disorganized urbanization, climate change, socioeconomic vulnerability, and environmental degradation [8,9,10].

In Brazil, CD affects between 1 and 3 million people, and most infected individuals are at the chronic stage of the disease [11]. It persists as a serious public health problem and is considered the fourth leading cause of death among infectious and parasitic diseases in the country, with an average of 4500 deaths per year [11,12,13]. In recent decades, it has shown changes in its transmission pattern; it was initially reported mainly in rural areas with vector transmission, and more recently, it has also been reported in large urban centers in Brazil, where it has arrived through different forms of transmission [12,14]. In addition, its importance lies in the magnitude of it morbidity and mortality, its high incidence, its wide distribution, and its impact on the Unified Health System (SUS), as well as its regional and national socioeconomic and social security implications [15,16,17].

Currently, the northern region of Brazil is considered endemic for CD, with the emergence of isolated vector-borne cases and the occurrence of family-level micro-outbreaks related to oral transmission through the ingestion of food contaminated with the infected vector’s feces or even the whole macerated insect [18,19,20,21]. Pará is the state with the highest number of cases, accounting for around 80% of its occurrence in Brazil [2,3,6]. In addition, the historical, socioeconomic, and environmental conditions of this state were determined by the pattern of occupation implemented in the region, which favored large development projects and reinforced the peripheral role of this territory as a producer of raw materials, marked by a high rate of deforestation, disorderly urban expansion, environmental degradation, and serious economic and social inequalities, which determined the establishment of socioenvironmentally vulnerable population groups with respect to disease risk factors, especially among rural populations [22,23,24,25,26,27,28].

Geotechnology is extensively used in health studies to generate information on various diseases and to help formulate and evaluate hypotheses about the spatial distribution of diseases by integrating vast amounts of cartographic, demographic, and epidemiological data [29,30]. Geotechnology tools make it possible to characterize diseases and their risk factors geographically, helping to tackle them in the different realities of the Amazon. With this in mind, the aim of this study is to analyze the relationship between the spatial distribution of CD and the environmental, socioeconomic, and public policy variables in three mesoregions of the state of Pará, from 2013 to 2022, with a view to contributing towards the implementation of public health policies that can tackle the disease in the Amazon.

## 2. Materials and Methods

**Figure 1:**
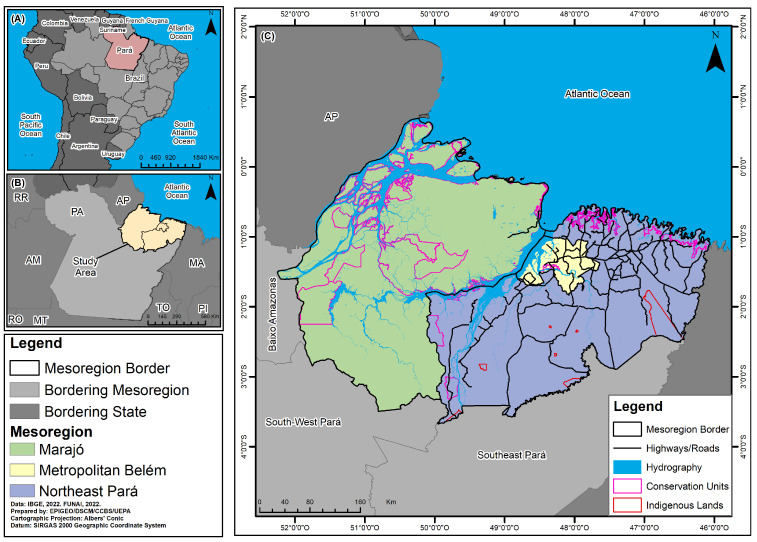
Spatial location of Pará [(**A**), red], the study area [(**B**), beige], and a map of the Marajó, netropolitan Belém, and northeast Pará mesoregions (**C**), Pará state, Brazil.

A descriptive, cross-sectional, and ecological study was carried out on all cases of CD recorded in three mesoregions of Pará state, included in the Notifiable Diseases Information System (Sistema de Informação de Agravos de Notificação—SINAN), made available by the Department of Epidemiological Surveillance at the State Health Department of the State of Pará, from 2013 to 2022. The spatial units of analysis for the study were three mesoregions in the state of Pará, namely, Marajó (16 municipalities/577,790 inhabitants); metropolitan Belém (11 municipalities/2,751,161 inhabitants); and northeast Pará (49 municipalities/2,023,064 inhabitants) (Figure 1).

Following an adaptation of the Miranda [31] methodology, epidemiological data (gender, age group, ethnicity, schooling, probable mode of infection, area of residence, and evolution) were obtained from the Ministry of Health’s Notifiable Diseases Information System (SINAN). Cartographic data (territorial limits, municipal seats, Indigenous lands. and conservation units), as well as demographic data (population numbers) and economic data (Gross Domestic Product—GDP), were obtained from the databases of the Brazilian Institute of Geography and Statistics (IBGE). The environmental databases related to land use and land cover used in this work (water, forest, forest plantation, natural formation, pasture, mosaic of uses, non-vegetated area, mining, and farming) were obtained from collection 9 of the MapBiomas project—Land Cover and Land Use, from the Climate Observatory’s Greenhouse Gas Emissions Estimation System, and those concerning the average monthly rainfall were obtained from the National Meteorological Institute.

The data obtained were debugged to remove inconsistencies and incompleteness and subsequently indexed in a Geographic Database (BDGeo) using Tabwin 415 software (Brazilian Ministry of Health, Brasília, FD, Rio de Janeiro, Brazil). The confirmed cases of CD were then georeferenced in the laboratory using a global positioning system (GPS) and then stored in the BDGeo. Fieldworks were made for environmental validation and terrestrial verification. To this end, we chose areas that had a significant number of cases in all of the mesoregions. For the descriptive and inferential analysis of the epidemiological data, absolute and relative frequency, as well as the adherence chi-squared statistical test, were used, with a significance level of 5%. Linear and polynomial trend analyses were carried out using the historical series (in Brazil, Pará, and the study mesoregions) and the seasonal series of prevalence with the average accumulated monthly rainfall (AAMR), respectively. All these analyses were carried out using the Bioestat 5.4 program (Mamirauá Institute, Brasília, FD, Brazil).

In the analysis of spatial distribution (direction and orientation), the prevalence and GDP of the studied mesoregions were presented on choropleth maps stratified by quantile in 5 categories (very low, low, moderate, high, and very high). In order to carry out the demand/supply flow analysis of access to treatment, the headquarters of the municipalities where patients lived and where they received treatment were identified and mapped. The flow map was constructed by calculating the distances between the seats of the municipalities of origin and destination, classified by quantile, with a distribution categorization that made it possible to stratify the distances into very close (up to 46.6 km), close (46.7 to 87.6 km), medium (87.7 to 155 km), far (155.1 to 256 km), and very far (256.1 to 506 km), represented by lines with color gradients in which the arrows indicate the final destination. All these analyses were carried out using ArcGIS 10.5.1 software (ESRI, Redlands, CA, USA).

Kernel density estimation was used to analyze the spatial distribution of the disease in order to identify possible clusters of cases stratified by quantile in 6 categories (absence, very low, low, moderate, high, and very high). To build the land use and cover map, the classes water, forest, forest plantation, natural formation, pasture, mosaic of uses, non-vegetated area, mining, and farming were considered. All these analyses were carried out using ArcGIS 10.5.1 software (ESRI, Redlands, CA, USA).

To assess the spatial autocorrelation between areas with human settlements (slums and urban communities) and with cases of CD, as well as the production of açaí with the prevalence of the disease, the bivariate local Moran’s index (I) was employed, using GeoDa 1.22 (Arizona State University, Tempe, AZ, USA). For this purpose, the hypotheses of “inverse” (I < 0), “random” (I = 0), and “direct” (I > 0) spatial autocorrelation were accepted, with significance determined at *p* < 0.05. Strong spatial autocorrelation was considered if (I) was close to one of the variation limits [−1, 1].

## 3. Results

**Figure 2:**
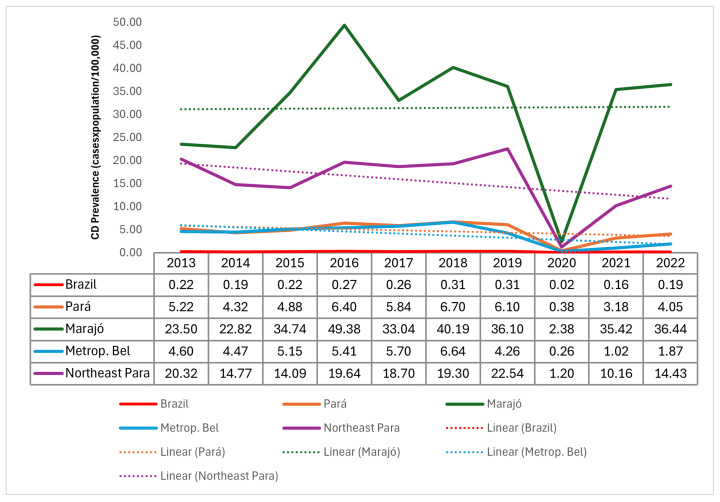
Historical series of Chagas disease incidence in the Marajó, Belém metropolitan, and Para northeast mesoregions from 2013 to 2022.

During the study period, 3664 cases of CD were confirmed in the mesoregions analyzed, with the northeast of Pará reporting the highest numbers (1817), followed by metropolitan Belém (926) and Marajó (921). The analysis showed a high number of cases of the disease in these mesoregions in relation to the state of Pará, with a significant reduction in 2020 (very low incidence) (Figure 2).

**Figure 3:**
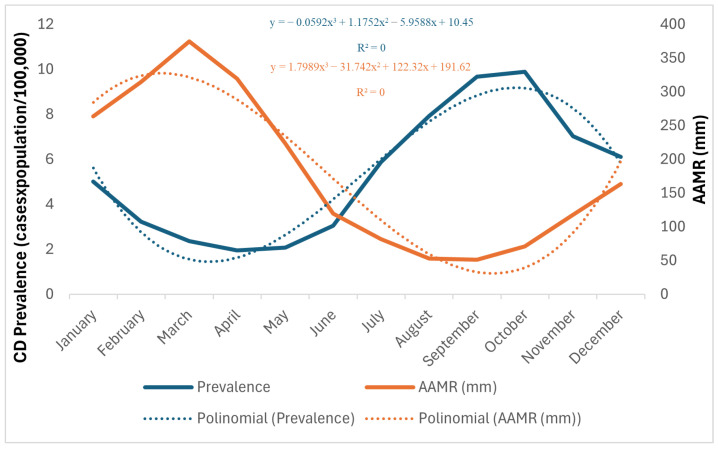
Historical series of monthly prevalence of Chagas disease and AAMR in the Marajó, Belém metropolitan, and northeast Para mesoregions from 2013 to 2022.

The epidemiological curve of monthly notifications during the study period showed a seasonal distribution pattern, with fewer cases and a downward trend in the first semesters of the series. However, there were significant peaks or increases in the number of cases in the second half of the year, especially from August to October (Figure 3).

The analysis of individuals diagnosed with CD in the study mesoregions revealed a higher occurrence among males, adults, and self-identified brown-skinned individuals, with possible oral transmission and a progression towards cure. Regarding the area of residence, the Marajó and northeast Pará mesoregions exhibited the highest number of cases in rural areas, whereas the metropolitan area of Belém reported the highest number in urban areas, all with statistical significance (Table 1).

**Figure 4:**
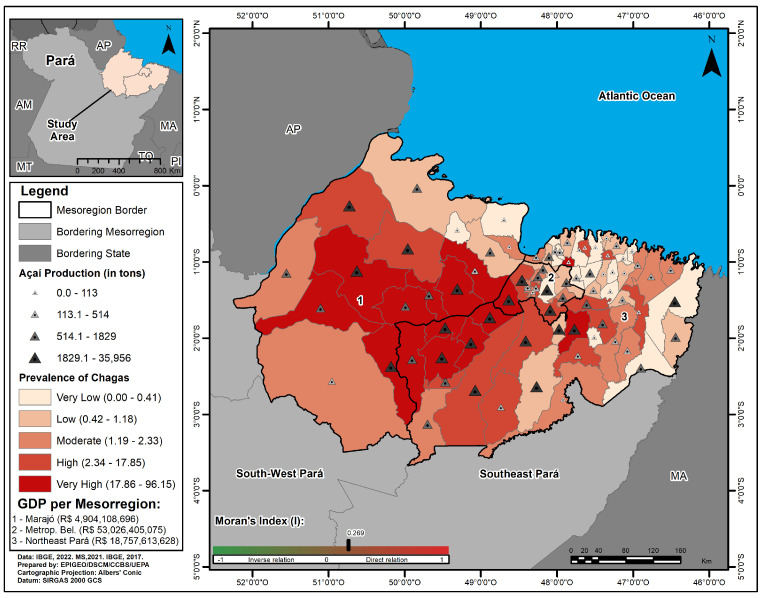
Map of Chagas disease prevalence, açaí production (in tons), and gross domestic product in the metropolitan mesoregions of Belém, Marajó, and northeast Pará, 2013–2022.

Analysis of the spatial distribution of CD cases in the mesoregions showed that Marajó had seven municipalities with a very high prevalence, two with a high prevalence, three with a medium prevalence, and two with a low prevalence. The metropolitan region of Belém had one with very high prevalence, three with high prevalence, four with moderate prevalence, and two with low prevalence. The northeast of Pará had seven municipalities with very high prevalence, ten with high prevalence, eight with moderate prevalence, and eleven with low prevalence. The production of açaí (*Euterpe oleracea* (von Martius, 1824)) in the municipalities and the GDP in the Mesoregions showed different values. The autocorrelation analysis between the disease prevalence and açaí production in the municipalities of the mesorregions studied, using the bivariate global Moran index (I), showed a moderately weak positive spatial dependency with I = 0.269 (*p*-value 0.0020) (Figure 4).

**Figure 5:**
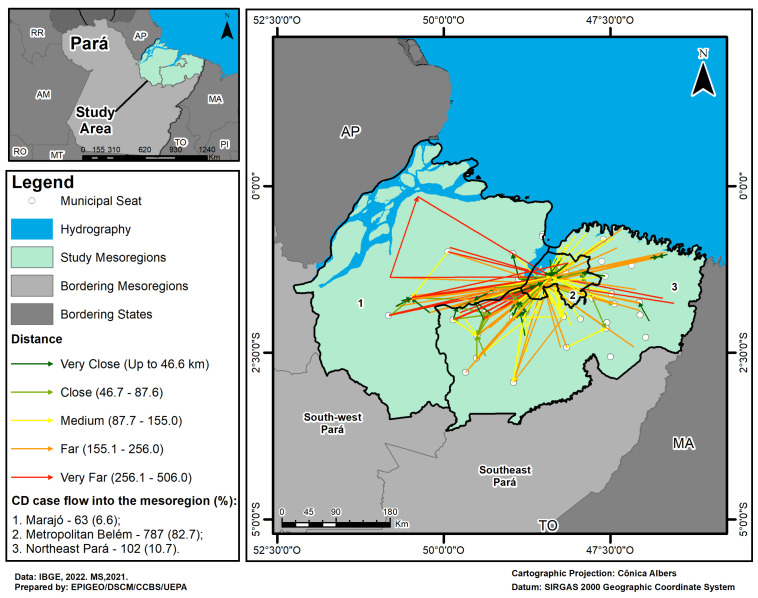
Map of the flow of patients with Chagas’ disease in the Marajó, Belém metropolitan, and Para northeast mesoregions, 2013–2022.

In terms of the geographical distribution of CD treatments, 537 patients living in the Marajó and northeast Pará mesoregions moved to the municipalities of Belém and Ananindeua in the Belém metropolitan mesoregion. These municipalities also act as a hub that attracts other municipalities within the same mesoregion (250 patients). The other flows are local, i.e., between municipalities within the mesoregion, and involve short distances covered and a small volume of patients. A significant number of patients (201) had their treatment funded by SUS, through Treatment Away from Home (TFD), and the use of services in another location as they live more than 150km from the place of care (Figure 5).

**Figure 6:**
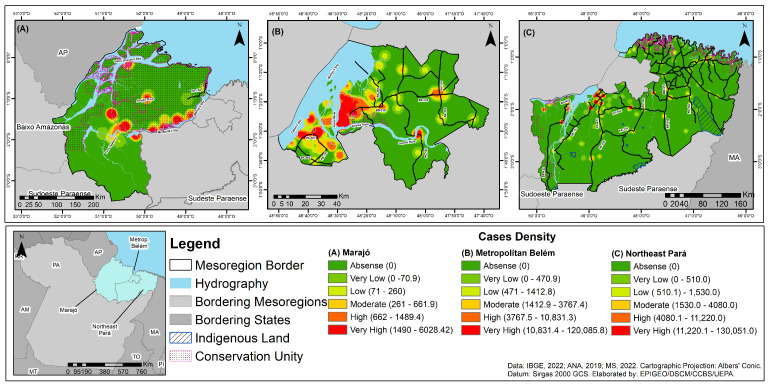
Density of Chagas disease cases in three mesoregions of the state of Pará, Brazil, for the period 2013–2022: (**A**) Marajó mesoregion; (**B**) Belém metropolitan mesoregion; (**C**) northeast Pará mesoregion.

Applying the Kernel technique revealed an inhomogeneous pattern of CD case distribution across the mesoregions. A very high prevalence of the disease was observed in all mesoregions, with evidence of vectorization in their municipalities (Figure 6). In the Marajó mesoregion, epidemiological clusters and corridors were identified that were formed by the municipalities of Ponta de Pedras, Muaná, São Sebastião da Boa Vista, Curralinho, Breves, and Anajás. At the same time, a similar event occurred in the northeast Para mesoregion in the municipalities of Moju, Igarapé-Miri, and Abaetetuba, located near the PA-475, PA-252, and PA-151 highways (Figure 6).

**Figure 7:**
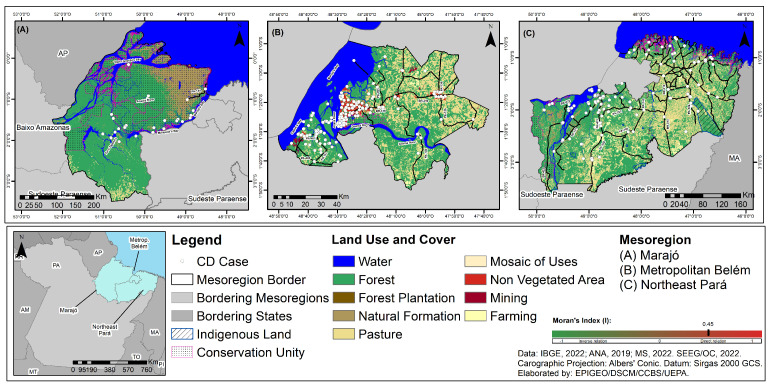
Spatial distribution of cases of CD, land use, land cover, and interpretation of the spatial autocorrelation of the Moran index (I) in the three mesoregions of the state of Pará, Brazil, for the period 2013–2022: (**A**) Marajó mesoregion; (**B**) Belém metropolitan mesoregion; (**C**) Pará northeast mesoregion.

The land use and cover map of the mesoregions studied shows that areas where deforestation has occurred due to various human-caused activities have also seen a large number of CD cases. A very high percentage of agriculture, pasture, mining, and urbanization was found, as well as the presence of small secondary forest fragments and isolated forest remnants in the Belém metropolitan and northeast Para mesoregions, with greater vegetation cover in the Marajó mesoregion. Clusters of cases were also observed in urban areas in the Belém metropolitan mesoregion, as well as in riverside communities in all the mesoregions studied, especially in the Marajó mesoregion. Moreover, cases of CD were observed in areas along roadsides, including around conservation units (UCs) and the northeast Pará indigenous land (TI Tembés and Anembés) (Figure 7).

The spatial analysis of the relationships between the areas of the mesoregions that reported cases of CD and those that had human settlements (slums and urban communities), using the bivariate global Moran index (I), showed significant direct and weak spatial dependency relationships between these variables, with I = 0.45 (Figure 7).

## 4. Discussion

The high occurrence of CD cases in the mesoregions studied during the period analyzed poses a significant public health challenge in the state of Pará and underscores the widespread nature of this disease. The epidemiological scenario observed highlights the ineffectiveness and inefficiency of intersectoral public policies adopted to date related to the control, monitoring, mitigation, and elimination of the disease. Furthermore, the significant reduction in notifications seen in 2020 may be related to the process of under-reporting diseases and illnesses of various etiologies caused by the health crisis brought on by the COVID-19 pandemic, according to studies carried out in other Brazilian regions [31,32].

The higher percentage of CD cases from August to October shows a seasonal pattern of occurrence associated with the interference of climatic conditions in the reproductive dynamics of triatomines during the dry season in these mesoregions [33,34,35,36]. This period is characterized by high temperatures, lower rainfall, and low relative humidity, as well as a higher frequency of forest fires [37]. Studies carried out on the relationship between these abiotic factors and the physiological process of CD vectors highlight the significant influence of temperature and humidity on increasing the number of blood meal, reducing the period of larval development, increasing population density and mating, dispersing populations and forming new colonies [38,39,40,41]. It is important to note that the period from August to November aligns with the açaí harvest, during which time there is a significant increase in its consumption and handling. This supports the theory of oral transmission through the ingestion of açaí contaminated by the pathogen [42,43,44].

The epidemiological profile of males and working-age adults with low education levels found in this study is recurrent in other research activities conducted in the Amazon region and Brazil and is related to the occupational exposure to disease risk factors and a lack of individual preventive practices, among other factors [33,34,35,36]. This scenario points to the need to strengthen inter-sectoral policies and programs that can optimize the implementation of actions and strategies to deal with the disease, since prolonged illness with CD causes changes in the family dynamics of these individuals due to different psychological, socioeconomic, and physiological factors, including the stigmatizing nature of the disease, time off work, serious systemic complications, as well as suffering related to the treatment of the disease associated with the risk of death [15,16,17].

The higher number of cases among brown-skinned people with low levels of schooling affected by CD reveals the ethnic–racial inequalities that have historically affected a large portion of the population in the mesoregions analyzed, given that education as a social indicator reflects the opportunities and inequalities that exist in the territory [45,46,47]. Thus, the results of the analysis highlight the impact of social determinants on the disease epidemiology, including poverty, low education levels, and increased exposure. In the study area, brown/black individuals living in environmentally deprived regions face higher risks of CD, lower wage opportunities, inadequate housing, lack of sanitation, and limited access to healthcare, illustrating a process of environmental racism [48]. The culmination of these factors makes it possible to establish the disease as a result of the interaction between the pathogen, the vector, and the socioenvironmental conditions in which the population finds itself.

The highest percentage of probable oral transmission of CD in the three mesoregions studied is associated with the consumption of regional foods, including açaí juice contaminated with *T. cruzi*. Studies carried out on this contamination emphasize its relationship with inadequate technical handling conditions during the harvesting, threshing, transportation, processing, storage, and final marketing phases of this product, including the following: 1—the presence of triatomine bugs in the treetops, which are collected from the bunches during harvesting; 2—the attraction of the bugs to the açaí fruit due to the heat and CO_2_ produced by intense respiration and fermentation after harvesting; 3—the organization of the baskets used for storing açaí in front of the houses of the extractivists, which are exposed to wild animals and vectors of the disease as they await the boat that will transport them to the consumer municipalities; 4—the attraction of the contaminated vectors by the light of the boats; 5—the handling of the açaí without adequate hygiene, pressing the insect between the seeds [49,50,51,52,53,54,55].

The discussion on oral transmission and food insecurity becomes even more pertinent when considering that açaí, the primary food involved, is a staple in the daily diet of the population and a nutritional supplement for many groups, forming a crucial part of the local cultural identity [18,20]. There are established guidelines in the regulatory framework to minimize contamination, such as Norm No. 1 of 7 January 2000 from the Ministry of Agriculture and Supply, which sets the standards for the identity and quality of fruit pulp to prevent food-borne disease outbreaks and health risks. However, many processing units in sociospatially and environmentally segregated areas lack adequate hygienic conditions and supervision [56]. This is particularly evident in some riverside areas where the fruit is repeatedly mashed by hand or with bottles in larger containers, i.e., without the necessary technical care to eliminate biological risks [57].

Another piece of evidence that highlights food insecurity and the inadequacy of culturally sensitive public health promotion policies is the significant number of cases of CD in the urban area of the Belém metropolitan mesoregion, which includes the state capital. Belém has a high consumption of fresh açaí (63.1 kg of fruit/inhabitant in the harvest and 22.5 kg in the off-season), which is higher than the consumption of dairy products (15.3 L per inhabitant/year) and beef (39.16 kg per inhabitant/year) [57,58], but part of the açaí consumed still comes from açaí producers who lack proper supervision. The urbanization process of the disease is linked to the disorganized growth of municipalities, which have numerous slums. This results in a belt of structural poverty, characterized by low economic status, inadequate environmental sanitation, poor housing conditions, and limited access to health services across the state [59,60].

The rural pattern of CD transmission in the Marajó and northeast Pará mesoregions highlights the region’s low economic development, which often obscures the visibility of social groups with distinct cultural perspectives compared to urban–industrial societies. These groups include subsistence farmers, extractivists, riverside dwellers, quilombolas, settlers, shellfish gatherers, traditional fishers, and Indigenous people [61,62]. These populations, with their specific territorialities, constitute the majority of the state’s peasant population. Particularly in the Marajó mesoregion, they reside in “forest cities” with distinct urban layouts and social organization intertwined with natural dynamics. Marginalized by state public policies for economic, social, and environmental development, they inhabit areas under environmental pressure and gradual degradation, making them more vulnerable to the spread of CD [63].

Because they live and work in and around the forest, participating in activities such as planting cassava and extracting fruit from palm trees native to the region, such as açaí and bacaba (*Oenocarpus bacaba* (Aublet, 1775)), these people cohabit and coexist with other species in this biome, close to the wild cycle of triatomines and their reservoirs. Furthermore, in recent decades, the various human impacts caused by the exogenous and hierarchical development of the Amazon have intensified the socioenvironmental vulnerability of these populations and influenced the establishment of vector disease transmission circuits in which abiotic and biotic elements interact [9,10,23,24,25,26,27,28]. From this perspective, it is urgent to implement public policies that include in their scope the mediation of actions and proposals related to One Health in order to understand this reality in a systemic and holistic way, considering the balance of the environment as the basis for maintaining life [64].

The association between the significant number of municipalities with very high and high prevalences of CD—which are classified as having high GDPs and are large producers of açaí on the regional, national, and international markets—observed in the mesoregions under study points to the need to review the conceptual purpose of the economic development plan for the state of Pará, in which local producers who carry out small-scale economic activities based on the use of renewable natural resources have a low financial return in relation to the process of marketing products that generate wealth and foreign exchange. In this regard, various factors, such as the economic accumulation of investor capital in the Amazon based on the appropriation of natural resources, the speculative retention of property associated with the inefficiency of technical support for land workers, the presence of middlemen in marketing, and the structural difficulties inherent in dealing with the state’s geographical barriers, contribute to the perpetuation of this vicious cycle of poverty–disease and, consequently, the serious epidemiological situation in these territories [55,65,66].

The aforementioned facts run counter to the first, third, and eighth Sustainable Development Goals of the 2030 Agenda, which advocate for the eradication of poverty and related diseases as a global challenge, as well as the need to ensure a healthy life with less economic inequality [66]. In addition, the epidemiological scenarios of CD observed in the municipalities of the Marajó Mesoregion, made up of municipalities with the lowest socioeconomic indicators in Brazil, point to the need to prioritize the implementation of integrative socioeconomic policies in Pará, differentiated and adapted to the municipal reality, which must have in their scope the guarantee of the basic rights of citizens as recommended by the Brazilian Constitution, which presents human dignity as central to social development, guiding the actions of the State [67].

On the other hand, the high percentage of cases that were cured suggests that the patients received adequate treatment and that the strategies employed by Primary Health Care to adhere to the protocol recommended by the Ministry of Health are showing satisfactory results. However, what is striking is the high percentage of cases in which the variable evolution and the area of residence of the patients and others were ignored. This fact indicates the presence of weaknesses in the notification system (municipal, state, and federal) related to the quality of the data collected in the municipality and directly affects the planning and execution of actions necessary to combat the disease, the health strategies to be adopted, the construction of indicators related to the epidemiological scenario, and the understanding of the territorial mobility of patients and the behavior of *T. cruzi* in various scenarios of social vulnerability [68].

The spatial analysis, using the flow map, showed that a large number of patients affected by CD had to travel to the municipalities of Belém and Ananindeua, which have most of the qualified physical and professional infrastructure for health services [69]. This is a major challenge for those who need medium- and high-complexity care in a state as large as Pará. In this sense, although the SUS guidelines advocate for a regionalized and hierarchical network in Brazil, the evasion of patients with serious clinical diagnoses of parasitic diseases to health regions located in other mesoregions points to care gaps, with divergences between planned and existing (real) regionalization, suggesting limitations in management designs related to network regulation and the treatment of cases [70,71], as well as the persistence of inequities associated with the unequal risk of falling ill and dying in the Amazon.

Particularly in the case of the Amazon region, an important factor to note in this social context is the travel distances imposed on patients treated away from home. These flows along roads and waterways, which involve medium and long distances, can make it difficult to provide adequate treatment in a timely manner and have a direct impact on the quality of life of those affected by the disease and their families due to various factors, including the difficulties of mobilization due to the particularities of the geomorphological formation of the mesoregions, which have areas of floodplain forests, channels, forest streams, rivers, and upland forest; the lack of paved roads and their vicinities; the inefficiency of the organization and spatial arrangements of the road and waterway routes; the need for treatment away from home; and the cost and time of travel from one’s municipality of origin to one’s final destination since this often requires several days of travel [72,73], especially in the Marajó mesoregion, characterized by major social problems, low coverage of health services, and a quantitative and qualitative deficit of professionals. The culmination of these facts highlights the geographical segregation of these individuals in terms of access to health services and limits the results achieved by egalitarian policies.

The Kernel technique demonstrated that the distribution of CD was not homogeneous in the mesoregions studied. The high and very high densities of cases of the disease located near rivers, highways, and in the central areas of the municipalities, including the formation of epidemiological corridors, highlights the process of spreading the disease in different environments, as well as the complexity of the dynamics of its vectorization, which involves factors such as the increased mobility of the population, the presence of untreated patients, and the circulation of infected animals without zoonotic control and changes in land use. In addition, these agglomerations may also be related to the occurrence of outbreaks of this disease with possible oral transmission, involving families and residents of the same neighborhood who report infection after consuming açaí containing *T. cruzi* [18,19,20,21,51,53]. These specificities confirm that health surveillance actions for CD must go beyond the border demarcations of the mesoregions since it is a public health problem related to the unplanned and unsustainable occupation of the territory.

The spatial analysis of the types of use and cover of land showed a direct relationship between the areas where CD occurs and those with changes in vegetation cover, especially for human settlements with the formation of slums and urban communities. The territorial dynamics of the mesoregions have given greater importance to economic growth and the production of goods, as well as the detriment of sustainable local development, environmental balance, and social equity. In this context, humans, as social subjects, by acting as a shaper of this Amazonian environment, have the altered ecotopes, ecological niches, and trophic chains of different living beings. This has had impacts on the intertwined parasitic networks of diseases of different etiologies, such as CD, and planners and policymakers have ignored the relevance of the unique physical–chemical and biological characteristics of the ecological balance of fauna, flora, and the physical environment, causing synergistic and cumulative impacts that have influenced the establishment of this disease [74,75].

These mesoregions have historically been used as a source of raw materials in an exploitative manner that left no results in terms of local development. In the Marajó mesoregion, land use and occupation has been influenced by activities linked to livestock farming, timber extraction, and subsistence agriculture. Meanwhile, in the metropolitan area of Belém, with the highest urban population concentration in the state of Pará, the municipalities of Marituba, Ananindeua, and Belém have 77.4%, 61%, and 52.4% of their populations living in slums, respectively. Finally, the northeast of Pará, characterized as one of the oldest deforestation frontiers in the Brazilian Amazon, is predominantly composed of pastureland, oil palm monoculture, and mining activities [46,59,63]. In general, the study area has a high level of socioenvironmental vulnerability, perpetuating the cycle of transmission of diseases of different etiologies, such as CD, since its municipalities emerged without proper planning to meet the demands of the land and economic structure, as well as the large development projects managed by national and international conglomerates [22].

The economic growth of these mesoregions has been marked by different opportunities for socioeconomic protagonism among their inhabitants, changes in the local population’s way of life, and the intense conversion of the primary forest into a mosaic of different types of vegetation and forms of anthropic land occupation. This accelerated and continuous deforestation has impacted the ecotopes, ecological niches, and the habitat of different species in the Amazon region [10,24,28,31]. In the specific case of CD vectors, when they left their natural environment in search of food and shelters appropriate to their physiological needs, they settled in peridomiciliary areas (pigsties, corrals, and chicken coops), being closer to the vegetation near homes and with the greatest possibility of contaminating food [3,4]. It is worth noting that it was observed on site that these insects are often used by children as “pull toys” in the municipalities of Breves and Soure and on the islands of Abaetetuba and Igarapé-Miri.

Finally, the occurrence of CD around Indigenous lands and PAs underscores the pressure of deforestation on these territories. It highlights the need to intensify efforts to promote and protect Indigenous health while also reflecting on the importance of this population group. With their historical ties to environmental protection and often undervalued political and identity configurations, they play a crucial role in the social, political, and economic formation of the mesoregions studied. In this sense, ignorance of this reality can be a complicating factor in drawing up public policies that respect the heterogeneity of traditional populations in the Amazon, contributing to their extermination.

## 5. Conclusions

CD remains a major public health problem in the study area. The epidemiological profile found is recurrent in other areas of the Amazon, which reinforces the importance of this study in the state with the highest percentage of cases in the region. The results of this study indicate the magnitude and potential for epidemic outbreaks of the disease as a result of interrelated biological, environmental, socioeconomic, cultural, political, and geographical risk factors, which make it possible to establish cycles of CD transmission in the mesoregions analyzed. In order to overcome this reality, it is essential to develop and implement more efficient and effective public policies that incorporate the specificities, singularities, and diversities that characterize the Amazon region, while respecting individuals and different population groups in their historical, political, environmental, and cultural contexts. Measures are needed specifically to mitigate the complex socioepidemiological scenario of CD that exists in the mesoregions studied, such as guaranteeing the basic rights of individuals; improving housing, health, income, education, food security, and basic sanitation conditions; and developing inclusive, permanent, systematic, and multi-level health education actions aimed at promoting One Health in all its dimensions. Furthermore, economic practices that prioritize social equity and respect for environmental limits in these territories must be implemented.

## Figures and Tables

**Table 1:** Epidemiological profile of Chagas disease in the Marajó, Belém metropolitan, and northeast Para mesoregions, 2013–2022.

Variável	Marajó		Metropolitan Belém		Northeast Pará		Total	
	n (%)	*p*-Value ^1^	n (%)	*p*-Value ^1^	n (%)	*p*-Value ^1^	n (%)	*p*-Value ^1^
**Gender**								
Male	514 (55.8)	0.0005	451 (48.7)	0.4498	961 (52.9)	0.0147	1926 (52.6)	0.0020
Female	407 (44.2)		475 (51.3)		856 (47.1)		1738 (47.4)	
**Age Group**								
Children	187 (20.3)	<0.0001	133 (14.4)	<0.0001	405 (22.3)	<0.0001	725 (19.8)	<0.0001
Adolescents	115 (12.5)		64 (6.9)		162 (8.9)		341 (9.3)	
Adults	541 (58.7)		619 (66.8)		1070 (58.9)		2230 (60.9)	
Elderly	78 (8.5)		110 (11.9)		180 (9.9)		368 (10.0)	
**Ethnicity**								
Brown Skin	818 (88.8)	<0.0001	717 (77.4)	<0.0001	1543 (84.9)	<0.0001	3078 (84.0)	<0.0001
Afro-Brazilian	29 (3.1)		42 (4.5)		93 (1.1)		164 (4.5)	
Caucasian	57 (6.2)		113 (12.2)		120 (6.6)		290 (7.9)	
Asian	4 (0.4)		1 (0.1)		9 (0.5)		14 (0.4)	
Indigenous	4 (0.4)		1 (0.1)		6 (0.3)		11 (0.3)	
Unknown	9 (1.0)		52 (5.6)		46 (2.5)		107 (2.9)	
**Schooling**								
Does not apply ^2^	63 (6.8)	<0.0001	49 (5.3)	<0.0001	172 (9.5)	<0.0001	284 (7.8)	<0.0001
Illiterate	42 (4.6)		8 (0.9)		49 (2.7)		99 (2.7)	
Primary school	386 (41.9)		268 (28.9)		735 (40.5)		1389 (37.9)	
High school	92 (10.0)		244 (26.3)		293 (16.1)		629 (17.2)	
Superior	19 (2.1)		90 (9.7)		70 (3.9)		179 (4.9)	
Unknown	319 (34.6)		267 (28.8)		498 (27.4)		1084 (29.6)	
**Probable Mode of Infection**								
Oral	520 (56.5)	<0.0001	435 (47.0)	<0.0001	609 (33.5)	<0.0001	1564 (42.7)	<0.0001
Vector	39 (4.2)		23 (2.5)		70 (3.9)		132 (3.6)	
Transfusion	2 (0.2)		1 (0.1)		4 (0.2)		7 (0.2)	
Accidental	1 (0.1)		1 (0.1)		2 (0.1)		4 (0.1)	
Transplacental	0 (0)		0 (0)		1 (0.1)		1 (0.0)	
Other	0 (0)		0 (0)		1 (0.1)		1 (0.0)	
Unknown	360 (39.1)		465 (50.2)		1130 (62.2)		1955 (53.4)	
**Evolution**								
Cure	815 (88.5)	<0.0001	792 (85.5)	<0.0001	1485 (81.7)	<0.0001	3092 (84.4)	<0.0001
Death by CD	4 (0.4)		12 (1.3)		12 (0.7)		28 (0.8)	
Death by Other Causes	2 (0.2)		10 (1.1)		11 (0.6)		23 (0.6)	
Unknown	100 (10.9)		112 (12.1)		309 (17.0)		521 (14.2)	
**Area of Residence**								
Urban	231 (25.1)	<0.0001	664 (71.7)	<0.0001	586 (32.3)	<0.0001	1481 (40.4)	<0.0001
Rural	468 (50.8)		180 (19.4)		908 (50.0)		1556 (42.5)	
Periurban	6 (0.7)		1 (0.1)		12 (0.7)		19 (0.5)	
Unknown	216 (23.5)		81 (8.7)		311 (17.1)		608 (16.6)	

n = Number of cases; ^1^ *p* < 0.05 (chi-square, adherence); ^2^ Children below school age.

## Data Availability

The data used in this study regarding socioeconomic and environmental variables can be found at https://cidades.ibge.gov.br/ (accessed on 2 August 2024) and (https://brasil.mapbiomas.org (accessed on 15 June 2024). All figures in this paper used public data and were prepared by the authors.
